# Evaluating RNA Structural Flexibility: Viruses Lead the Way

**DOI:** 10.3390/v13112130

**Published:** 2021-10-22

**Authors:** Connor W. Fairman, Andrew M. L. Lever, Julia C. Kenyon

**Affiliations:** 1 Homerton College, University of Cambridge, Cambridge CB2 8PH, UK; cf496@cam.ac.uk; 2 Department of Medicine, Cambridge University, Level 5, Addenbrookes’ Hospital (Box 157), Cambridge CB2 0QQ, UK

**Keywords:** RNA structure, RNA flexibility, RNA viruses, SHAPE, proximity ligation, NMR, SAXS, smFRET

## Abstract

Our understanding of RNA structure has lagged behind that of proteins and most other biological polymers, largely because of its ability to adopt multiple, and often very different, functional conformations within a single molecule. Flexibility and multifunctionality appear to be its hallmarks. Conventional biochemical and biophysical techniques all have limitations in solving RNA structure and to address this in recent years we have seen the emergence of a wide diversity of techniques applied to RNA structural analysis and an accompanying appreciation of its ubiquity and versatility. Viral RNA is a particularly productive area to study in that this economy of function within a single molecule admirably suits the minimalist lifestyle of viruses. Here, we review the major techniques that are being used to elucidate RNA conformational flexibility and exemplify how the structure and function are, as in all biology, tightly linked.

## 1. Introduction

RNA is a critical, central component of the chemical processes of life, not just as an information storage mechanism but also as a spatially dynamic ligand and chaperone, and in some cases, catalytic, molecule. As is common in biology, form and function are inseparable, and the physical properties of RNA are what enables it to perform such a diverse range of roles. Viruses pack functionality into small genomes, and often use the different properties of RNA to carry out distinct parts of their lifecycle. The most familiar of the structural properties is the ability to form Watson–Crick base pairs; this facilitates the generation of RNA duplexes which are significantly stiffer and less deformable than DNA duplexes [1]. This additional stiffness also translates to RNA secondary structures, with RNA hairpin elements requiring more force to disrupt them than equivalent DNA duplexes. Conversely RNA itself is much more labile than DNA due to its additional 2′ hydroxyl group on the ribose allowing cyclization and hydrolysis [2]. The duplex length and the base composition of the helix both influence the flexibility and strength of interaction [3]. There is often a degree of nucleotide bias in viral genomes; for instance, the HIV-1 genome has a high density of A’s and a consistently low proportion of C’s, in contrast to deltaretroviruses, which are A-poor but C-rich [4]. This has implications for the secondary structure of viral RNA, as well as influencing codon usage and amino acid diversity [5].

Besides the classical canonical base pairing, RNA has many well-categorised non-canonical interactions that allow for structural diversity. Each base has three edges which can hydrogen bond to other bases: the Watson–Crick edge, the Hoogsteen edge, and the sugar edge. These alternative interactions include the G-U wobble base, which has similar bonding energy to an A-U pair and can fit within a Watson–Crick helix [2]. Having the ability to form G-U as well as A-U or G-C pairs adds to the ability of the same RNA sequence to form multiple different structures. Other bases such as pseudo-uracil, inosine, and dihydrouridine can also be incorporated [2]. Additionally, more than one edge can be interacting at any one time, allowing the formation of triple bases such as C-G-A where guanine is involved in both a canonical and sheared base pair [6]. Common non-canonical interactions centre around G-A pairs due to their hydrogen bonding capabilities allowing multiple arrangements of the two purines. There are six dominant modes of G-A base pair interaction [6]. Each of these interactions can bestow subtle differences to the characteristics of the base pair, which in turn affect the RNA secondary structure; for instance, the sheared G-A arrangement stiffens an internal loop more than an imino pair. It has also been demonstrated that transitions between different ionic bonding arrangements can occur in a smooth pathway, hinting that RNA conformational flexibility may be facilitated by these transitions [6].

With such a plethora of possible interactions, and the ability to transition between different states, it is no surprise that higher order RNA structure is incredibly diverse and is often flexible. However, an increasing amount of evidence suggests that these secondary and tertiary structures are comprised of a modular combination of conserved motifs (examples are described in Table 1) [7], with adenine appearing to be more commonly present in structure cores and junctions [8].

It is unclear whether motif evolution is convergent or divergent, but such motifs are present in a wide array of organisms [9] and combine to generate the RNA fold [8]. The use of motifs plays a role not only in static conformations but also in controlling flexibility of the RNA, the 3WJ motif of *Bacillus* phage phi29 packaging RNA, for instance, is highly thermodynamically stable [10].

Many RNAs exist as ensembles of various conformations, usually with a dominant ground state along with rarer excited state structures, and the composition of the surrounding environment can tune the ensemble composition. Interactions with ligands can induce conformational changes in RNA, a well characterized example being that of bacterial riboswitches, such as the SAM-1 riboswitch family. As is typical of riboswitches, the SAM-I riboswitch senses and responds to levels of a particular metabolite (SAM: s-adenosyl methionine, indicative of cellular methionine levels), which causes multiple changes in the RNA structure of an expression platform, hindering translation. Remarkably, the SAM-I*_Xcc_* riboswitch was recently observed to respond both to the presence of SAM and to the presence of uncharged initiator methionine tRNA, which triggers the opposite structural change, leading to methionine production [11]. Viruses, to our knowledge, do not use metabolites to control RNA structural switches in this manner, but they do use similarly complex RNA structural switches.

**Figure 1:**
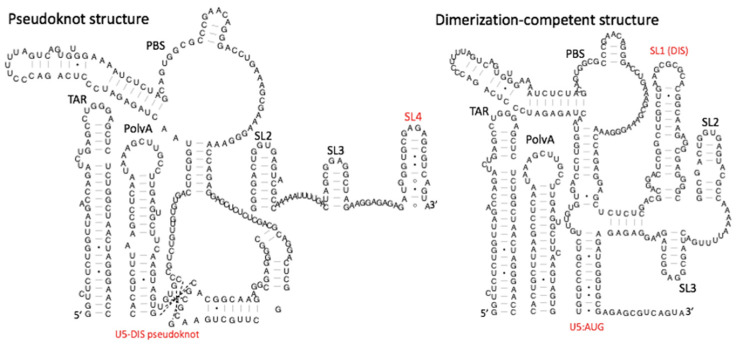
The HIV-1 genomic RNA leader contains a structural switch. Left hand panel: pseudoknot structure, where the DIS palindrome forms a pseudoknot with upstream U5 sequences. Right hand panel: the same U5 sequence pairs with the Gag start codon to form the U5:AUG helix. Structures that change during the switch are labelled in red. Structures are drawn according to data from Kenyon et al., 2013.

Mg^2+^ binding to RNA affects the stability of conformations and variations in its concentration can induce structural changes, such as the more tightly folded three helix junction present in 16s RNA being favoured at higher Mg^2+^ concentrations [12]. Chemical modification of nucleobases can also affect conformation; N^6^-methylated adenosine (m^6^A) is a common modification in viral as well as eukaryotic RNAs. It destabilizes A-U base pairing and can have stabilizing or destabilizing effects on RNA duplex formation depending on where in the structure methylation occurs [13]. The flexibility of RNA structures is crucial for their regulatory function, especially where it involves interactions with proteins. The HIV-1 TAR (shown in Figure 1) hinge region, for example, adopts a particular conformation within the ensemble in order to bind its ligand with high affinity, in a mechanism known as conformation capture [14]. Studies of RNA recognition motif regions of polypyrimidine tract binding protein (PTPB) also support this theory with the pyrimidine motif, containing a C*syn*U*anti* arrangement, performing a “conformational readout” to recognize its RNA target [15].

Viruses may represent the peak of complexity when it comes to their RNA structure, since there is enormous selection pressure for the economical use of their small genomes to maximise replicative fitness. An interesting example of the structural importance of RNA in the virus lifecycle can be seen in the Φ29 bacteriophage prohead RNA (pRNA). Here, the 174nt non-coding pRNA self-assembles into a nano-ring which acts as a scaffold for the recruitment of ring ATPase and, hence, the completion of the bacteriophage packaging motor used to condense the viral genome into the nucleocapsid [16]. Mutational disruption of stem loop D flexibility resulted in the inhibition of nano-ring self-assembly, showing the importance of the structural fluidity necessary to perform this role. In many viruses, the job performed by pRNA in Φ29 is effected by a protein, but the fact that RNA alone can achieve the same result speaks to its power as both a structural and functional molecule.

Coding functions of viral RNA also make use of RNA secondary structure, providing a dual use of information storage and regulation. For instance, the first open reading frame (ORF) of the Severe Acute Respiratory Syndrome Coronavirus 2 (SARS-CoV-2) full-length RNA contains a pseudoknot that facilitates the ratiometric translation of two overlapping sequences, ORF1a and ORF1b, through ribosomal frameshifting [17]. A similar method of frameshifting in retroviruses also precisely controls the ratio of Gag to GagPol coding transcripts.

## 2. Analysis of RNA Structure and Flexibility

The ability of RNA to adopt many conformations has made structure probing technically challenging. The earliest studies focused on solving two-dimensional secondary structures by chemical and enzymatic techniques complemented by computer-based free energy minimization and phylogenetic conservation data. Static three-dimensional models were later derived by techniques such as crystallography and NMR and global folds subsequently gleaned from lower-resolution methods such as SAXS and Cryo-electron microscopy. Developments in all of these techniques have meant that they all are still capable of providing valuable complementary information and although the challenge of solving individual structures from within structural ensembles is still significant, advances are being made. No single method has all the answers yet, but the combination is starting to reveal some of the unsuspected complexity of viral RNA conformational versatility.

### 2.1. SHAPE and Other Secondary Structure Probing Techniques

**Figure 2:**
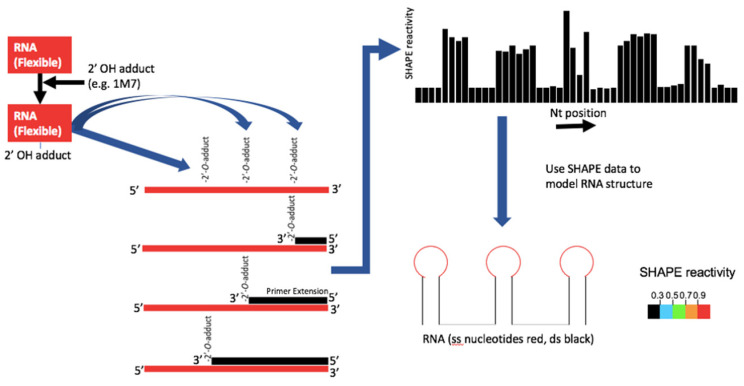
Process diagram for high-throughput SHAPE structure determination. The 1M7 reacts covalently with a 2′OH on the sample RNA. This occurs preferentially where the backbone is flexible. Reverse transcriptase generates a family of cDNAs of length corresponding to the sites of 1M7 adduct formation. Comparison with a sequencing ladder allows readout of amount of cDNA product of each length. This ‘SHAPE reactivity’ is used to inform minimal free energy prediction software.

RNA secondary structure probing typically has used enzymatic (ribonuclease) or chemical reagents that react with specific bases according to their chemical reactivity and/or the structure in which they are found in the polyribonucleotide chain. Modification of the base or cleavage of the chain is detected using reverse transcription as the reverse transcriptase (RT) enzyme dissociates from the template at these sites, giving cDNAs of varying lengths in different proportions. These data are then used to identify single- and double-stranded regions of the RNA and can inform minimal free energy structural prediction algorithms. This was typically a laborious process that necessitated the use of multiple probing reagents and radiolabelled RNA electrophoresed on long acrylamide gels. SHAPE (selective 2′OH acylation analyzed by primer extension, Figure 2) [18] revolutionised RNA secondary structure probing for two reasons: firstly, the SHAPE reagents can acylate nucleotides agnostically of the actual base and thus theoretically provide a measure of the single or double strandedness of every nucleotide. Secondly, the methods used to analyze the cDNAs have improved.

Fragment analysis of cDNA lengths by capillary sequencing has enabled high-throughput analysis. ‘High-throughput SHAPE’, which uses capillary electrophoresis and fluorophore-labelled cDNAs, enabled the study of the entire HIV-1 genome inside virions [19] as well as its 5′ region in different biological states [20], concluding that the HIV-1 5′UTR is relatively conformationally stable. Such analyses, however, report upon a structural average, and modifications to the technique have since helped to separate and probe individual conformers, elucidating a plethora of structural changes in retroviral 5′UTRs. ‘In-gel SHAPE’ separates conformers by native gel electrophoresis before probing them individually; such experiments showed conclusively that a large-scale structural rearrangement occurs in the HIV-1 RNA upon dimerization [21]. Modifications to the original SHAPE reagents have extended the range of reagents possessing different half-lives, which has allowed scientists to follow RNA structural changes across timescales of milliseconds to minutes, as well as to work within cells and virions [22]. Retroviral virions bud from the cell in an immature, noninfectious state, subsequently maturing by protease cleavage and structural rearrangements. The use of time-resolved SHAPE and a protease deficient murine leukaemia virus mutant showed that the immature virion contains a specific structural intermediate in the genomic RNA dimerization pathway that is distinct from the structure inside mature virions [23].

The development of SHAPE-MaP, which uses an RT that skips a nucleotide at the site of the bound reagent rather than terminating the reverse transcription, has enabled the use of next-generation sequencing and smaller amounts of viral RNA template [24]. In SARS-CoV-2, it has shown that some regions of the genome contain stable RNA structures and that others show low correlation of SHAPE reactivities between experiments, hinting perhaps that slightly different cellular conditions effect structural changes [25]. In dengue (DENV), SHAPE-MaP has shown that upon packaging, RNA undergoes a structural rearrangement, which may be brought about and stabilized purely by the capsid protein or may be due to an RNA structural switch, and that the dominant RNA structure of the genome inside virions is in the circularized form [26]; this is perhaps surprising given that the circularized form is thought to begin replication, which happens early in the viral lifecycle after cell entry.

Modifications that increase the half-life of SHAPE reagents in an aqueous environment have enabled the cellular phases of viral lifecycles to be examined. In a study comparing all four dengue serotypes with four geographically distinct strains of Zika virus (ZIKV), a remarkable degree of RNA structural conservation was observed not only between strains of the same virus but also between the different flaviviruses [27]. The structures formed were different inside cells and in virions, with less RNA structure forming inside cells, likely reflecting the need to facilitate translation of the genome.

SHAPE has also been coupled with photo-crosslinking, to separately probe RNA structural changes and protein binding sites, using both to build a picture of how the viral RNA changes structure upon binding a protein ligand. When this technique, known as ‘XL-SHAPE’, was applied to the dimeric HIV-1 5′ leader RNA interaction with Gag, extensive structural changes became apparent, indicating remodelling of most of the structural elements in this 350 nt region [28].

SHAPE-related techniques are not without their limitations, however, as they are reliant upon structural modelling algorithms to fit the data to the structure, and they do not give direct evidence of specific nucleotide pairings. The reactivity of nucleotides to the SHAPE reagents can be influenced by factors other than simple base pairing. There are further confounding problems, including that the longer half-life needed to penetrate cell membranes and probe RNAs inside cells also allows binding of reagents to nucleotides that can interconvert between two or more structures, effectively marking these regions as ‘single-stranded’ rather than being involved in switching between alternative base pairings. Some authors have specifically noted that structural conservation assessed by intracellular SHAPE scores can be lower than the sequence conservation between viral strains [29], which may reflect this phenomenon.

### 2.2. Proximity Ligation

**Figure 3:**
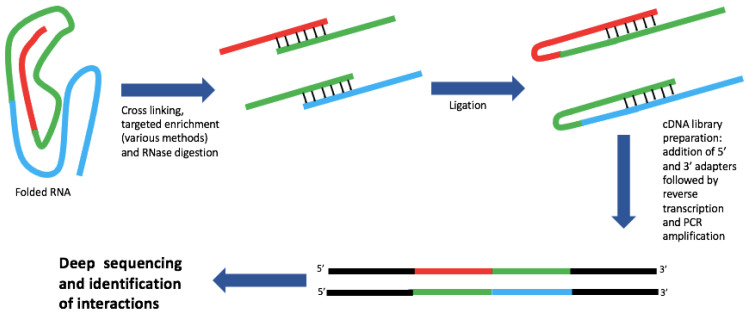
Process diagram for proximity ligation. Although the specifics of different proximity ligation methods vary, many follow the general scheme shown above. The modular nature of the scheme allows a mixing and matching of different steps to suit the experimental goals.

Proximity ligation detects spatially interacting nucleotides and provides direct evidence of interactions rather than making predictions using mapping software (Figure 3). Initially adapted from methods to detect RNA–protein binding sites such as cross linking and immunoprecipitation followed by sequencing (CLIP-seq) [30], a range of novel techniques for determining RNA structure has emerged, and been used to study viral RNA–RNA and viral-host RNA–RNA interactions (Table 2). The general mechanism for such techniques is broadly similar: cross linking is followed by targeted enrichment (if desired), limited digestion by RNase, RNA–RNA proximity ligation, cDNA library generation and finally sequencing. A prominent advantage of such a general scheme is the modularity of the different steps allowing customization of protocols to suit a particular experimental need.

Psoralen-based proximity ligation techniques have become particularly important in recent years as they do not require a pull-down step of RNA–RNA interacting with a protein. Psoralen is a plant-based intercalating agent which forms reversible covalent bonds with RNA–RNA duplexes following UV irradiation; derivatives of psoralen such as AMT have increased cell permeability characteristics [31].

**Table 2:** Proximity ligation techniques.

Technique	Distinguishing Features	Reference
Psoralen cross-linked, ligated, and selected hybrids (PARIS)	Uses 2-dimensional gel electrophoresis to enrich cross linked fragments	[32]
Sequencing of psoralen cross-linked, ligated, and selected hybrids (SPLASH)	Biotin-streptavidin enrichment	[33]
Ligation of interacting RNA (LIGR)	RNase digestion enriches fragments	[34]
Dual crosslinking, immunoprecipitation, and proximity ligation (2CIMPL)	Enriches fragment using magnetic bead tethering via an anti-NP antibody, also contains an additional cross-linking step before introduction of AMT	[31]
Cross-linking of matched RNAs and deep sequencing (COMRADES)	Biotin-streptavidin enrichment	[35]
RNA proximity ligation (RPL)	Omits psoralen cross linking step, instead relying on spatial proximity of bases in RNA secondary structures to facilitate chimera formation	[36]

Many of these techniques have been used to interrogate viral genomic RNA structure; PARIS, SPLASH, and COMRADES have all been used to investigate Flaviviruses. Such studies revealed that certain RNA secondary structures remain conserved across different ZIKV lineages, particularly within the 3′UTR, and identified a unique long-range interaction between the 5′UTR and the E protein coding region in the Asian lineage that is linked to increased infectivity [29]. Long-distance base pairing between 5′ and 3′ cyclization elements has long been known to mediate a conformational change in flavivirus genomes during replication and interactions with human miRNAs were also found which may be important for regulating viral RNA conformation as well as impacting miRNA activity [35].

Proximity ligation techniques can illustrate structural flexibility in the RNA by the identification of nucleotides that have binding partners in two or more regions. Perhaps some of the most powerful evidence of conformational flexibility of RNA comes from these experiments, as 48.8% of long (≥500 bases) and 41.6% of short (<500 bases) pair-wise interactions form alternative structures in both DENV and ZIKV genomes [27]. In some regions of the genome such as the 5′UTR, at least three conformational changes occur during the lifecycle [29]. The use of proximity ligation has particular pertinence to the study of segmented RNA viruses as it is able to identify conclusively intrasegmental interaction sites, which are important for viral assembly in particular. 2CIMPL mapping of influenza RNA identified multiple intrasegmental interactions, with particular hotspots that appear to interact with multiple sequences in multiple different segments, such as ones within the nucleocapsid protein (NP) RNA segment. This technique also revealed that synonymous base changes in these hotspot regions can result in genome-wide intersegmental interaction changes without a loss of replicative fitness, suggesting that influenza has evolved to have flexibility in the RNA interaction networks and structures it uses. Mutations in the NP hotspot resulted in new hotspots emerging in the polymerase (PA), haemagglutinin (HA), and neuraminidase (NA) segments. Such insights are vital for understanding influenza genome reassortment and mutation (and thus pandemic preparedness) [31], and have also been shown using SPLASH, with particular insights into the limitations on successful strain reassortment [37]. The likely transient nature of some of these RNA–RNA interactions during the viral lifecycle makes the choice of technique important: 2CIMPL proved to be more successful than LIGR/PARIS at identifying intersegmental interactions (RNA hybrids occurring within 1.14–1.21% of mapped reads compared to 0.004%) thanks to the addition of a second crosslinking step to preserve spatial information of the vRNP segments.

### 2.3. NMR

Certain nuclei, such as ^1^H, have spin, and when placed in an electromagnetic field, some of them are promoted to the higher energy state, where they are spinning antiparallel to the field. The behaviour of such nuclei when they return to the lower-energy state (‘relax’) is governed by their chemical environment and can be measured in NMR experiments. Nuclei influence one another in this respect along covalent bonds via spin–spin couplings and through space via the Nuclear Overhauser Effect. The elucidation of flexible RNA structure via NMR should, on the one hand, be more suitable than the use of X-ray crystallography since it does not rely on the formation of static crystal structures. However, RNA has relatively low proton density and a limited chemical diversity of its monomers. The resonances associated with it also often have short relaxation times, leading to difficulties in solving spectra associated with RNAs larger than around 70 nt. NMR has thus typically been used to solve solution structures of small RNAs, and has produced vital insights into the structural flexibility of individual stem-loops and their roles in viral lifecycles. NMR studies on multiple RNA structural elements from SARS-CoV-2 recently allowed comparison of their structural stabilities, as imino resonance line widths differ between static or metastable helices. The 5′UTR structures SL2 and SL3 were observed to vary, with the highly conserved SL2 forming a stable helical structure and SL3, which contains the TRS (transcription regulating sequence) as a metastable structure [38]. The TRS presumably needs this structural flexibility as during transcription it pairs with sequences in the developing negative strand to control where strand switching occurs, and thus which subgenomic RNA is made. When it does this, it forms different RNA structures with each switch region in the genome. The stabilities of these structures in turn has been proposed to control the balance of subgenomic RNAs and hence the abundance of individual proteins that are produced [39].

Isotope substitution has extended the range of NMR to much longer molecules. This has perhaps been demonstrated most clearly in the case of HIV-1 RNA genomic dimerization. The stem-loop that mediates dimerization, SL1 (a bulged helix loop shown in Figure 1), does so via a 6 nt terminal kissing-loop interaction that extends into an intermolecular interaction between the subtending helices, known as the extended duplex. This was shown by adding ^15^N-edited RNA at an equimolar ratio with unlabelled RNA, to distinguish between inter- and intramolecular interactions [40]. NMR also revealed that a 100% conserved 3-1 bulge within the SL1 helix was highly metastable, likely facilitating unwinding [41]. More recently, ^2^H editing has been used similarly to the ^15^N editing study above, upon the whole 5′ leader RNA, to show, quite remarkably, that the intermolecular pairing upon dimer maturation extends much more widely beyond SL1, to involve the U5:AUG helix [42]. As this helix has been shown to be important for genome packaging, this raises the distinct possibility that the extent of intermolecular interaction between the dimers helps to control the HIV-1 encapsidation process. Another modification to NMR, using ^13^C-enriched fragments ligated to unlabelled RNA, has allowed the study of individual regions within much larger RNAs. This was instrumental in identifying a structural switch that occurs between two distinct structures of the HIV-1 leader RNA: U5 nucleotides involved in the U5:AUG helix pairing can alternatively form a pseudoknot structure with the dimerization initiation site on SL1 (Figure 1). This switch has been proposed to control the viral switch from translating to packaging its genomic RNA [21,43], as shown in Figure 1, where the pseudoknot structure exposes the AUG in a relatively flexible stem loop (SL4, a 21 nt structure with a G-A bulge) and the dimerization-competent structure exposes the dimerization initiation site, on SL1.

### 2.4. Sm-FRET

**Figure 4:**
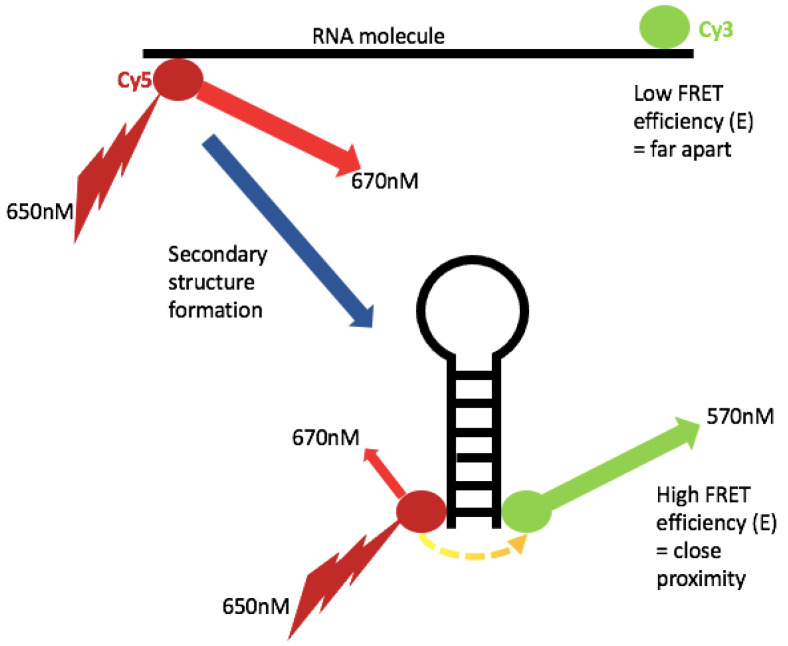
Principle of single molecule FRET (sm-FRET). sm-FRET relies upon the spatial proximity of a donor and acceptor dye. Where the dyes are far apart, there is little resonance energy transfer and a low *E* value; conversely, where the dyes are much closer to each other, transfer efficiency is greatly increased. Emission from the acceptor dye is a measure of proximity.

**Figure 5:**
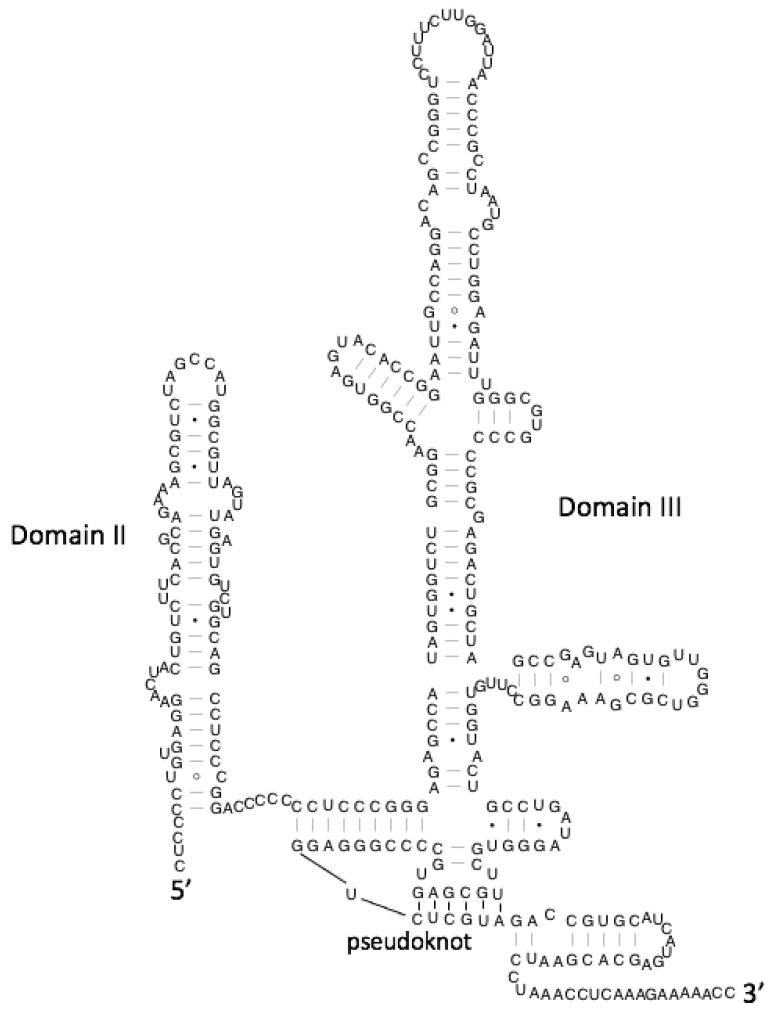
Structure of the HCV IRES. The two major domains, II and III, are highlighted, alongside the pseudoknot.

Single-molecule Förster Resonance Energy Transfer (sm-FRET) measures the spatial proximity of a donor and acceptor fluorophore and thus determines the distance between the molecules to which they are attached. This has obvious applications for studying the conformations of RNA molecules, and was used to solve the core RNA structure within telomerase [44]. Several groups have applied sm-FRET-based methods to study viral RNA [45,46,47,48,49]. FRET relies upon energy transfer from an excited donor dye to an acceptor dye; quantification of the brightness of both donor and acceptor leads to a ratiometric calculation of FRET efficiency (E), which is a reliable indicator of proximity (Figure 4) [50]. Sm-FRET is usually used in cases where knowledge of absolute distances is not necessary since conversion of E to distance is dependent upon the environment and orientation of the dye; however, control experiments with each dye can provide good estimates [50]. A viable dye must be of sufficient brightness and photostability; cy3 and cy5 are commonly used, but of particular relevance to this review is dy547, which can replace cy3 in custom RNA constructs [50]. The detection of fluorescence is most commonly carried out by camera-based total internal reflection fluorescence microscopy (TIRFM), with time resolution being determined by the frame rate of the camera [50,51]. Reduction of instrumental noise is crucial in FRET, and here, TIRFM has an advantage over confocal microscopy (which can also be used) by reducing the excitation volume to a thin layer above the sample solution [51]. TIRFM also enables high-throughput protocols, by exciting a large surface area thousands of molecules can be sampled in tandem.

sm-FRET has revealed the conformational dynamics of the Hepatitis C virus (HCV) internal ribosomal entry site (IRES) (Figure 5) [45].

Within the HCV IRES, domain II controls, amongst other things, the progression from initiation to elongation. In the absence of ligand, it sits in a tightly bent conformation, proposed to act as a brake on the ribosome. Upon ligand binding, the domain straightens and elongates without changing its secondary structure. Such a large-scale tertiary structural change without an effect on secondary structure is proposed to be a hallmark of viral RNA structural switches substantiated by further experiments on IRES structures from a range of viruses. HCV IRES domain II has also been studied in complex with 40s ribosomal RNA using smFRET, revealing that domain II exists in two conformational states even whilst in complex, instead of the previous single conformation model [52]. It has also been proposed that HCV must undergo a multistep rearrangement during complex formation with the 40s ribosomal subunit, highlighting the key role of gRNA flexibility during initiation and maintenance of the complex.

Like other flaviviruses, West Nile Virus (WNV) cyclizes its RNA before genome replication in the host and has structurally complex 5′ and 3′ UTR regions to facilitate this. One of the necessary 3′UTR cyclization signals is concealed within a metastable 3′ helix, which, in human cells, requires AUF1 p45 binding to mediate a structure switch exposing the cyclization signal. sm-FRET was used to show that this switch can also be mediated in the mosquito host, by the mosquito squid proteins p30 and p32, although less efficiently than in humans. smFRET also showed the RNA switch to be temperature sensitive, acting as a ‘thermometer’ for the virus [47]. Such RNA structural changes at different temperatures could be advantageous for viruses that can cause temperature fluctuations in their mammalian hosts, and in particular for arboviruses, which often replicate at a lower temperature in the insect host than in the mammalian host. Highly conserved Flavivirus dumbbell structures present in the 3′UTR are also implicated in genome cyclization and have been investigated by sm-FRET [46]. Part of the dumbbell forms a pseudoknot with a 3′ sequence, occluding this 3′sequence and preventing it from cyclizing with the 5′UTR. This structural prevention of cyclization is proposed to enable translation. The dumbbell region in Donggang (DONGV) virus was observed to heavily favour a pseudoknot conformation, with transient unfolding followed by rapid reformation highlighting the low kinetic energy barrier of its formation. As with many tertiary RNA structures, pseudoknot formation was dependent upon a higher Mg^2+^ concentration (10 mM). Further mutational analysis then revealed that the ability of the P1 and P2 helices to bend into a close conformation, present in a four-way junction motif on the opposite side of the dumbbell structure, affected the frequency of formation of the pseudoknot [46].

Although smFRET has most commonly been used to follow structural changes in real time, it can also be used to model tertiary RNA structures, if the secondary structure is known. This was carried out for the HIV-1 packaging signal RNA (a highly structured stretch of 150–250 nt in the 5′ UTR, containing several conserved stem loops centred on SL1, SL2, and SL3 (Figure 1), revealing the presence of two very different structural populations, presumably representing monomer and dimer. Occlusion of the dimerization initiation site in the RNA using a complementary locked nucleic acid reduced this to a single structural population and enabled modelling [49].

### 2.5. SAXS

Small angle X-ray scattering (SAXS) provides a cost-effective, time-efficient method to investigate molecular structure dynamics in near-native conditions. The technology itself is not a recent development, having been used to assess tRNA structure in the 1960s [53]; however, improvements in predictive software have greatly increased the value of the technique. SAXS does not provide single-atom detail as its maximal resolution is around 2 nm [54], but samples can be measured in solution without the need for labelling or crystallization, and the conformational dynamics of molecules can be observed over a temporal or conditional range [55]. The principle of SAXS relies upon an X-ray beam scattering as it passes through a sample; a background reading of scattering through the solvent alone acts as a control and the remaining signal provides a SAXS curve. Software packages can then infer structural characteristics about the samples, such as using Kratky plots to determine the extent of folding [53] and electron pair distance distribution function (PDDF) to find real space measurements and hence molecular shape [55]. A problem for this, as with many other structure determining techniques, is the ability to distinguish between simultaneously existing conformers of the same molecule, as often the multiple signals obtained from the many coexisting species can merge into an average which can lead to incorrect structure prediction. Since RNA conformers are known to frequently exist in ensembles, it is important that they can be distinguished from each other. Ensemble fitting, developed by Bernado et al., uses software to generate all possible structures within the conformational space, and then selects a mixture of these that best fits the available data [56].

The ability of SAXS to detect flexibility within a molecule makes it an obvious choice to study viral RNA dynamics in their native state. Both the poliovirus and rhinovirus interactions with poly-C binding protein (PCBP) have been investigated using SAXS [57,58]. The rhinovirus 5′ UTR contains a type I IRES and a smaller 83 nucleotide 5′ clover leaf structure (5′CL) that consists of one stem (SA) and three stem loops (SLB, SLC, and SLD). PCBP is able to bind to both the IRES and SLB of the 5′CL and plays a role in both replication and translation. Only when PCBP is bound to the IRES can the virally encoded 3C protease cleave it, switching from viral translation to replication [57]. SAXS data combined with NMR data showed that the 5′CL structure was Mg^2+^ dependent, adopting perpendicular helices or a compact arrangement of SLB and SLD, which are both proposed to be important at different stages in the replicative cycle, depending on the appropriate conditions [57]. In an analogous situation in poliovirus, which also uses a type I IRES, the RNA structure changes to a more flexible, base accessible one upon PCBP cleavage. The binding target of PCBP, stem loop 4 (SLIV), is a complex structure within itself and contains 4 smaller stem loops (a, b, c, and d). Additional analysis by cryoEM determined two structural classes of PCBP/SLIV complex, suggesting a degree of flexibility around its four-way junction. Taken together, the techniques show the presence of flexibility of SLIV both in and out of complex. Both Hepatitis B (HBV) and HCV have also been investigated using SAXS [59,60]. SAXS data helped to support an NMR structural determination of the 6nt HBV priming loop, in which the partially stacked G16 and U17 were identified as potential residues responsible for initiating DNA synthesis [59]. HCV encodes a noncoding 3′ domain known as 3′X which contains a 16nt palindromic genome dimerization signal that partially overlaps with a 7nt sequence that forms a long-range interaction with a complementary site within the ORF. Both structures formed are thought to be vital to the lifecycle, controlling replication, translation, and possibly genome packaging. SAXS was used to characterize the 3′X domain structure at high and low ionic strength, forming monomers and dimers, and enabling insights into the dimerization process. The use of SAXS in this context also suggests that a large-scale structural rearrangement is necessary in order to mediate the 3′X-ORF interaction [60].

Long non-coding sub genomic RNAs are produced by several species of flavivirus and are of functional significance particularly in controlling host antiviral responses. SAXS analysis has shown that their structure is modular and that differences between them can be used to explain mechanistic variations [61]. Some of these structural modules are similar across different flaviviruses, such as the xrRNA1-2′s, which are compact in solution, indicating reduced flexibility. However, small differences in pseudoknot formation in one of the dumbbell structures in dengue virus lead to a markedly different tertiary structure from West Nile and Zika [61]. The HIV-1 Rev response element (RRE, a highly conserved ~350 nt *cis*-acting sequence within the *env* open reading frame) and its interaction with Rev has also been analyzed using SAXS. Multiple Rev molecules must bind to the RRE, and this nucleoprotein structure engages the Crm1 nuclear export pathway and enables transport of partially spliced and unspliced HIV RNA out of the nucleus. Using SAXS, a structural rearrangement has been suggested to occur in the RRE as each successive Rev molecule binds, eventually exposing a cryptic Rev binding site that enables further nucleation of Rev [62,63]. Sherpa et.al. have also previously used non-denaturing polyacrylamide gel electrophoresis to show that the RRE exists as two conformers, containing either four or five stem-loops, that confer different rates of viral replication [64].

### 2.6. Other Techniques

Although SAXS, NMR, sm-FRET, SHAPE, and proximity ligation are the most commonly used techniques to study viral RNA dynamic structures, many other techniques have also been used to complement them, such as cryogenic electron microscopy (cryo-EM). Here, samples are immobilized within a carbon (or sometimes gold) nano-grid using vitrified ice and images are then captured by a transmission electron microscope before software packages combine the different orientations of the complex captured into a robust three-dimensional model. Cryo-EM has been applied to viral RNA in several studies, and usually accompanies another technique that can capture the RNA dynamics in a near-native environment (such as SAXS or sm-FRET). Limitations of cryo-EM include that is has a low resolution, with structure determinations below 100 kDa being rare [65], and highly flexible RNAs can inhibit particle classification [61]. Cryo-EM was able to successfully determine two different conformations of the poliovirus IRES:poly (rC) binding protein 2 (PCBP2) complex as well as capturing the RNA alone, confirming its predicted cruciform structure as well as hinting at a degree of flexibility in sub stem loop c (SLc). Cryo-EM has also been used to investigate the cricket paralysis virus (CPV) type 4 IRES interaction with the 40s and 80s ribosomal complexes, where a degree of density fragmentation of the A-rich variable loop (VRL, termed variable due to differences at the loop apex between strains) present in complex with the 40s but not 80s suggested structural rigidity unique to the former [48]. The HCV IRES capturing an actively translating ribosome has also been imaged using cryo-EM: surprisingly, the IRES is able to initiate cap-independent translation without inhibiting the ongoing cap-dependent translation, which actually enhances translation of the viral RNA [66].

A field of study that high-resolution cryo-EM is particularly useful for is imaging the RNA packaging structure within the nucleocapsid of viruses. Asymmetric reconstructions of Brome mosaic virus (BMV) and MS2 bacteriophage have been completed and revealed differing levels of RNA packaging [67]. Analysis of BMV at 3.9 angstrom resolution showed that the capsid protein interacts with the gRNA at the two- and threefold vertices, with the RNA forming different conformations. Apart from this, the genome remains largely disordered. This contrasts with the MS2 genome, where 80% of nucleotides can be individually resolved (multiple conformers still exist, however) [67], implying a much higher degree of structural order to the RNA within the virion in the latter.

Smaller genomes can sometimes be structurally solved using X-ray crystallography, which showed that in tobacco mosaic virus (TMV), RNA structure inside the capsid is beautifully ordered and exhibits a high degree of symmetry but does not contain the motifs known to be necessary for viral replication, translation, or RNA transport, indicating that a genome-wide conformational change must occur when it is released into the host cell [68].

Gel-based experimentation methods have also been used to elucidate viral RNA structural interactions via mutational analysis. The wheat yellow mosaic virus genome segment RNA1 contains a 5′ UTR IRES and a 3′-cap-independent translation element (CITE). In line cleavage analysis in the presence and absence of the 3′ CITE followed by electrophoretic mobility shift assays (EMSAs) identified a structural switch that controls translational activity [69], via the formation of a small number of G-C base pairs between discontinuous sequences in either helix 1 or helix 2 and the intervening linker region. Interactions between helix 1 and the linker inhibit translation, whereas those between helix 2 and the linker enhance translation. These interactions are mutually exclusive as the tertiary structure generated by one attenuates the activity of the other [69]. The use of in-line probing also hinted that plant RNA viruses generally maintain weaker, more flexible RNA structures in their IRES elements than do animal RNA viruses.

An emerging technique that is particularly useful for studying long non-coding RNAs (lncRNAs) is analytical ultracentrifugation (AUC). Using the sedimentation velocity (SV) and sedimentation equilibrium (SE) modes can give information about molecule size, purity, aggregation, hydrodynamic shape, and ensemble composition whilst requiring very small sample sizes compared to other biophysical techniques [70]. AUC is beginning to be used as a reliable quality control step for assessing RNA integrity before proceeding with more laborious techniques such as SAXS, as demonstrated by Mrozowich et al. when interrogating flavivirus non-coding regions. The AUC data supported urea PAGE results suggesting that one of the RNAs had a degree of degradation, with all four of the RNAs being tested showing a minor second peak that could correspond to multiple conformational states [71].

Several forms of spectroscopy are also emerging as viable techniques for determining RNA structure. Circular dichroism makes use of the differing absorbance between left and right circularly polarized light, with different biomolecules displaying different absorbance spectra signatures. Having been applied widely to DNA and proteins the technology is now beginning to be applied to RNA, thanks to advances in synchrotron capabilities allowing the spectral range to become as low as 170 nm [72]. Down at these short wavelengths, charge transfer regions can be seen, giving insights into the RNA structure and dynamics in solution across a wide temporal and environmental area. It is thought that this technique could complement NMR in the future. Another form of spectroscopy, near-infrared (NIR) Raman spectroscopy, is also proving useful. It provides information about the energy modes of vibrating molecules subject to modulation by the polarizability of adjacent nuclei [73]. The technique has only become viable with the latest instrumentation but allows for even dilute solutions to be reliably characterized under near-native conditions, and indeed, the avocado sunblotch viroid genome has been analyzed this way [73].

An interesting strategy of investigating RNA excited states (ES) using 2′-O-methyl (Nm) modification of the ribose sugar has recently been characterized. Nm modification has been known to alter the biological activities of RNA and is used endogenously by many organisms. Abou Assi et al. demonstrated that Nm achieves this by altering the conformational ensemble composition, usually by increasing the incidence of ES conformations that base pair the modified residues compared to the dominant ground state (GS) conformation [74]. These ensemble shifts can be detected using NMR or UV optical melting, which can give insights into the kinetics of conformation state transition states, a highly relevant parameter for those wishing to study RNA dynamics. The strategy also holds promise for RNA design, since the ability to modulate RNA conformation without changing the base sequence facilitates an additional layer of complexity.

Alongside the wet lab techniques to study RNA structure and conformational change, computational structure prediction techniques are also applied extensively. Many such programs center around the philosophy of finding a local energy minimum, using simplified ‘coarse-grained’ molecular models and optimizing algorithms such as Ant colony or Metropolis to refine initially random arrangements into an optimized structure. The many ensuing 2D prediction programs, such as ACOfoldpath and NARES-2P [75,76], are continually compared for accuracy via the CompaRNA server [77]; some algorithms, such as SimRNA, which predicts 3D as well as 2D structures, analyze conformational landscapes and thus provide information on RNA structural flexibility [78]. Although the challenges of 3D modelling of RNA are substantial, much has been learned from the RNApuzzles consortium, who have been blind testing a range of computational and experimental predictions before comparing the outcomes with crystal structures. The results have shown that accurate prediction requires a combinatorial approach of de novo prediction techniques and comparison with previously solved structures and motifs in databases [79]. Machine learning has the potential to greatly enhance the accuracy of computational RNA structural prediction; however, in the meantime, experimental techniques continue to be vital.

## 3. Summary

The armamentarium of techniques used to study RNA structure is growing, and with it, a greater appreciation of the extraordinarily versatility of this molecule and its ability to carry out multiple roles simply by using conformational variants of a single molecular species. The molecular environment is clearly critical and complicates interpretations of many current methods that rely on analysis in non-native conditions. As with much of molecular biology, pivotal findings in this field have been made studying viral RNA, although, paradoxically, these may represent some of the most densely compacted overlapping functional entities that exist in RNA molecules. Better in vivo techniques are emerging and alongside these is the rapidly expanding field of nucleotide modification of RNA and its effect on RNA structure and function. Enhanced methods of RNA sequencing such as nanopore are starting to make these more easily analyzable. Combined with computational advances and the emerging power of machine learning, the future of RNA structure/function analysis has an exciting but unpredictable future.

## Figures and Tables

**Table 1:** Common RNA structure motifs.

Motif	Characteristics
Three-way junction (3WJ)	The junction of three helices.
Four-way junction (4WJ)	The junction of four helices.
Kink-turn	Helix–internal loop–helix motif with a 50° bend in the helical axis. Two classes, N1 and N3.
C-loop	Increased helical twist.
Right angle motif	Internal helical angle of 90°.
Stem loop	Intramolecular helix with a terminal loop of three to eight nucleotides connecting the two sides of the stem. Tetraloops, where four nucleotides are in the loop, are particularly common, with UNCG and GNRA being especially stable.
Paranemic motif	Crossover motif of stacked helices
Kissing loops	Base pairing between two helix loops.
Ribose zippers	Hydrogen bonding interaction between the 2′OH groups on ribose sugars of unpaired, anti parallel bases.
Cross strand purine stacks	Internal loop motif where the six membered rings of two purines stack across two strands, as opposed to classical same strand stacking
Bulge-helix-bulge	4 bp A-form helix with a 3 bp bulge either side.

## Data Availability

Not applicable.
